# The Utility of Multistate Models: A Flexible Framework for Time-to-Event Data

**DOI:** 10.1007/s40471-022-00291-y

**Published:** 2022-06-29

**Authors:** Jennifer G. Le-Rademacher, Terry M. Therneau, Fang-Shu Ou

**Affiliations:** Division of Clinical Trials and Biostatistics, Mayo Clinic, 200 First Street SW, Rochester, MN 55905 USA

**Keywords:** Multistate models, Survival analysis, Time-to-event data, Competing risks

## Abstract

**Purpose of Review:**

Survival analyses are common and essential in medical research. Most readers are familiar with Kaplan–Meier curves and Cox models; however, very few are familiar with multistate models. Although multistate models were introduced in 1965, they only recently receive more attention in the medical research community. The current review introduces common terminologies and quantities that can be estimated from multistate models. Examples from published literature are used to illustrate the utility of multistate models.

**Recent Findings:**

A figure of states and transitions is a useful depiction of a multistate model. Clinically meaningful quantities that can be estimated from a multistate model include the probability in a state at a given time, the average time in a state, and the expected number of visits to a state; all of which describe the absolute risks of an event. Relative risk can also be estimated using multistate hazard models.

**Summary:**

Multistate models provide a more general and flexible framework that extends beyond the Kaplan-Meier estimator and Cox models. Multistate models allow simultaneous analyses of multiple disease pathways to provide insights into the natural history of complex diseases. We strongly encourage the use of multistate models when analyzing time-to-event data.

**Supplementary Information:**

The online version contains supplementary material available at 10.1007/s40471-022-00291-y.

## Introduction

Time-to-event data (also known as survival and failure time data) are commonly collected in multiple disciplines including medicine, epidemiology, environmental health, engineering, operations research, and physics. These data provide information on whether the events of interest (e.g., death, dementia, disease recurrence) occurred and when those events occur for each subject. Classical analysis methods for time-to-event data are the Kaplan–Meier estimator and Cox proportional hazard models [1, 2]. These methods are adequate in studies where there is only one type of event of primary interest.

When there are multiple events of interest, the aforementioned methods may not provide a full picture of the relationship. In these situations, methods that can elucidate the underlying relationship between the covariates, the intermediate outcomes, and the outcomes of interest are needed. Multistate models, first mentioned by Cox and Miller in 1965, provide a flexible and broader framework to extend familiar methods [3]. The fundamental theory for multistate models was established using the counting process methodology [4•]. Detailed guidance has appeared more recently along with practical software [5–7].

This paper provides a brief introduction of multistate models and highlights their utility using examples from published papers on this topic. Specifically, we will introduce terminology of multistate models, demonstrate the construction of a state space, and describe important quantities that can be estimated from a multistate model. We will wrap up the paper with annotated references of selected publications on multistate models for readers who are interested in deeper knowledge on this topic.

## Multistate Model–Terminology and State Space

A multistate model is a framework that uses continuous time processes to describe and model subjects’ experiences over a time course [4•, 8]. All multistate models consist of two essential components: the state(s) and the transition(s). “State” is the time-varying/longitudinal status of a subject at a given time. “Transition” is a directional movement from one state to another. A state can be transient or terminal. A state is considered transient if a transition from that state to another state is possible; whereas a state is considered terminal (also called absorbing) if transition from that state to another state is not possible—i.e., once a subject enters a terminal state, s/he is assumed to remain permanently in that state. An absorbing state can be biological, such as death, or it can be due to research interest, e.g., in a competing risk model, all states (other than the initial state) are absorbing.

**Fig. 1:**
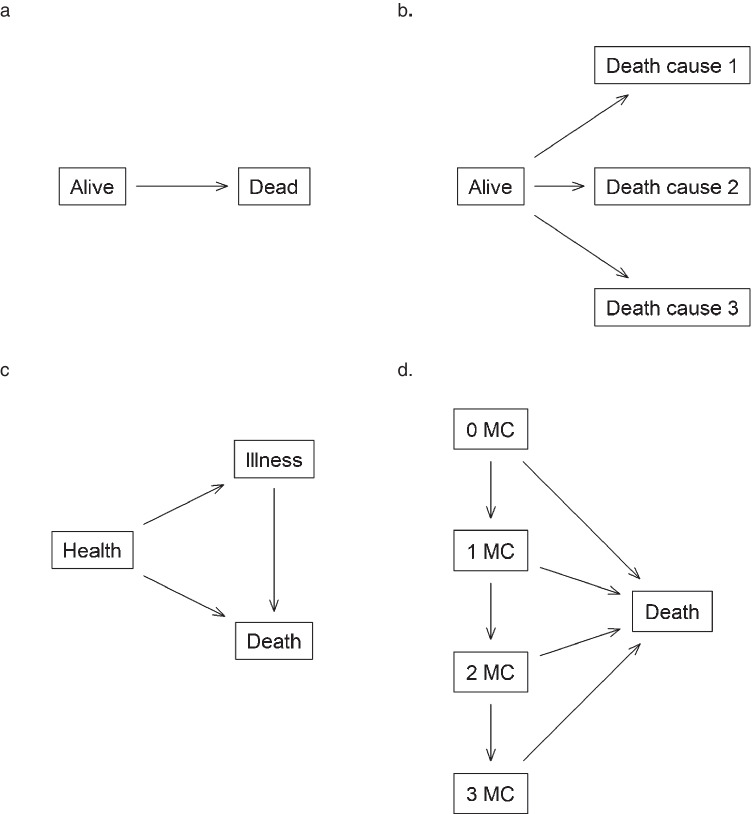
A collection of state space. **a**. Two-state mortality model, **b**. Competing risks model, **c**. Illness-death model, **d**. A state space for progressing from 0 to 3 metabolic comorbidities and death. MC: Metabolic comorbidities

A “state space” is a graphic depiction of the possible states and transitions of a multistate model. It is an essential visualization tool when planning design and analysis of time-to-event data. Figure 1 shows a collection of four state space. Figure 1a is the simple survival model, Fig. 1b corresponds to a competing risks model, Fig. 1c is an illness-death model, and Fig. 1d is a more complex model showing comorbidity progression associated with nonalcoholic fatty liver disease (NAFLD) which will be used as an example later in this manuscript. In each figure, boxes are the states and arrows represent potential transitions between states. A state with both input and output will be transient, a terminal (or absorbing) state is one that has no further transitions.

Because multistate models are flexible and can accommodate a wide range of scenarios, it is important to construct multistate models that have the appropriate level of complexity to address the scientific question(s) of interest and yet simple enough for clinical interpretation and for the model to be reliably estimated. Specifically, when constructing a multistate model, include only the states and/or transitions that are necessary to answer the research questions. Not all possible states and transitions need to be included. For example, in a study where patients can experience both relapse and death, the following models can be considered depending on the goals of the study:

- If the primary focus is on death regardless of disease relapse, then the simple survival model (Fig. 1a) may suffice;
- If the focus is on the risk of relapse or death (without relapse), then a competing risks model (Fig. 1b) would be appropriate; or
- If the primary goals focus on the progression from complete remission to relapse to death, then an illness-death model (Fig. 1c) may be needed.

In another example, Allen et al. were interested in the natural history of NAFLD and the progressive burden of 0, 1, 2, or 3 metabolic comorbidities (MC) with death as a competing risk (Fig. 1d) [9]. However, if they were only interested in the disease process from having no comorbidity (Health) to having at least one metabolic comorbidity (combining one, two, and three comorbidities into one single state, i.e., illness) and death, then the simple illness-death model (Fig. 1c) would suffice. On the other hand, if they were interested in the disease process from having no comorbidity to specific comorbidities then to death, a more complex state space can accommodate the research need (Supplemental Fig. 1); however, the analysis results may be more difficult to interpret and some transitions may have very few patients.

Another point to consider when constructing a multistate model is when the number of subjects experiencing a certain transition is very low (e.g., ≤ 5), that transition may not be modeled reliably.

## Multistate Model – Statistical Analysis

In this section, we illustrate analyses of time-to-event data using multistate models starting with descriptive summary using the non-parametric Aalen-Johansen estimator followed by advanced modeling of the hazard rates using Cox-type models.

### The Probability of Being in a State at a Given Time

The simplest estimate, and the one we will almost always start with, is the probability of being in any given state at a given time *t*, which can be represented as a vector *p(t)* with one element per state. Just as the survival probability at a given time in a mortality model (Fig. 1a) can be estimated using the Kaplan–Meier estimator, the probability of being in a certain state at a given time in a multistate model can be estimated using the non-parametric Aalen-Johansen estimator, the multistate analog to the Kaplan–Meier (both the Kaplan–Meier estimator and the cumulative incidence are special cases of the Aalen-Johansen estimator) [1, 10]. The Aalen-Johansen estimates of the states in a multistate model can be plotted over time in a similar manner to the Kaplan–Meier curves. These curves show the likelihood of a subject being in one of the states over time.

**Fig. 2:**
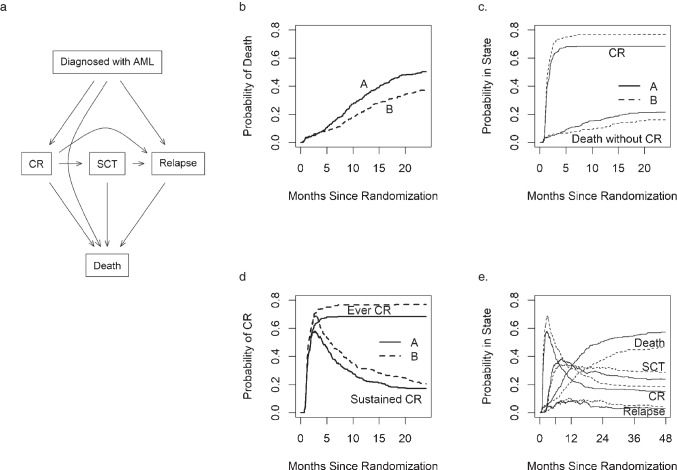
The Myeloid data example. **a**. State space in consideration for the Myeloid dataset, **b**. Overall mortality by treatment arms, **c**. CR and death without CR as competing risks, **d**. Ever in CR (competing risks) vs. Sustained CR (illness-death model), **e**. Myeloid dataset, all four states. Diagnosed with AML: Time of Randomization; CR: Complete Response; SCT: Stem-Cell Transplant; A: Control Arm (solid line); B: Experimental Arm (dashed line)

We illustrate the Aalen-Johansen estimate using the Myeloid data example from the survival package of R [6, 11, 12•]. Patients with newly diagnosed acute myeloid leukemia (AML) were randomized to receive an experimental or a current standard of care (SoC) for induction chemotherapy. If the chemotherapy is effective, the disease burden is expected to reduce to a very low, undetectable level (termed complete response, CR). Patients may then go on to receive a hematopoietic stem cell transplant (SCT) to achieve long lasting remission. Unfortunately, there is no long-term cure to the disease. Figure 2a shows the state space of a conceptual model of the Myeloid data example. The actual observed patterns; however, are more complex (Supplemental Table 1); there are, for instance,106 patients who received SCT without achieving CR.

Figure 2b–e show four separate sets of Aalen-Johansen curves for the Myeloid data. As overall survival was the primary endpoint of this study, we first looked at the curves depicting the overall mortality by treatment arms (Fig. 2b), which shows that the experimental treatment (B) has a lower overall mortality rate compared to SoC. To understand the impact of treatments on disease burden, we examined the curves in Fig. 2c which show the probability of complete response (CR) and death before CR as competing risks. The CR curves show a nearly identical CR rate over the first 2 months, with addition late CRs in arm B while the mortality without CR curves begin to separate after 5 months since randomization, showing a lower rate of death without CR in the experimental arm (B), suggesting that there might be a direct connection between the experimental treatment and the occurrence of late CR events. One question of interest in this study was whether the CR was likely to sustain. Figure 2d shows the curves for the CR state from an illness-death model with three states: diagnosed with AML, CR, and relapse/death where CR was treated as a transient state (i.e., patients experienced CR can transition to relapse/death); overlaid with the CR curves from the prior competing risks model (Fig. 2c) where CR was treated as an absorbing state. We see that those achieved CR in both arms appear to have similar durability, which gives yet another hint that the late CRs of arm B may be “as good as” earlier CRs. Figure 2e shows the evolution of all 4 states over time. It shows for instance that relapse is quickly transient, i.e., there is never a large fraction of patients in that state at a given time.

Note that the curve for participants remaining in the “Diagnosed with AML” state is omitted from the figure because it is simply one minus the total probabilities from all other states at a given time which is low and is not of interest in this example. Analogous to the Kaplan–Meier estimates, the Aalen-Johansen estimates are unadjusted, i.e., they do not account for other covariates.

Multistate Aalen-Johansen curves provide a useful tool for understanding the evolution of patients through a set of states and it should be the first step for any multistate analysis. Plots such as Fig. 2e that show all the states can be overwhelming, especially if curves for all states are included by covariates, e.g., imagining Fig. 2e that would include 4 Treatment*Sex curves for each state, one of the challenges is how to best display results using colors, line types, multiple panels, etc.

#### Absolute Risk: Other Measures

From the Aalen-Johansen estimates, two other clinically relevant measures can be obtained: the average time spent in a given state (also known as the mean time-in-state or the sojourn time) and the expected number of visits to a given state (sometimes known as the lifetime risk).

Mean time-in-state is estimated by calculating the area under the probability-in-state curves generated from the Aalen-Johansen estimates. Returning to the study by Allen et al., using this approach they found that the mean lifetime of subjects with nonalcoholic fatty liver disease (without cirrhosis) was 4 years shorter than their age- and sex-matched controls [9]. They further found that these patients spent about 75% of their remaining life in comorbid condition.

In situations where it is not possible to follow all subjects until their terminal event due to limited resources or where the events of interest are expected to occur quickly and the research questions focus on this shorter period, the time-in-state can be estimated restricted to a shorter time, i.e., termed restricted mean time in state. In the case of the simple survival model (Fig. 1a), the restricted mean time in state is more commonly known as the restricted mean survival time (RMST). Le-Rademacher et al. used a multistate model on a clinical trial dataset enrolling patients with AML to model transitions among four states (diagnosed with AML (i.e., time of randomization), first complete remission, disease relapse, and death) [13]. In this trial setting, since most of the events are expected to occur within 48 months from randomization, the focus of the time spent in each state was restricted to 48 months from randomization. Therefore, the interpretation of the time spent in a state in this example is the average amount of time a patient spent in that state in the first 48 months after randomization. They found that patients treated with the experimental therapy stayed in first complete remission 3–8 months (depending on their biomarker) longer than those receiving placebo. In patients with low ratio internal tandem duplication biomarker, the experimental treatment also prolonged the time alive in relapse by 2.3 months compared to placebo. The experimental treatment was also associated with a longer life expectancy of around 3 months over the 48 months study window. This analysis provides more granular understanding of the treatment effect through various intermediate events that occurred between randomization and death, complementing the results of the clinical trial primary analysis [14]. Time spent in a state is well defined and interpretable even when a state can be visited multiple times or visited in various order.

In more complex multistate models, certain transient states can be visited multiple times. For example, in the model with state space illustrated in Fig. 2a, a patient can experience multiple remissions (first, second, or third), multiple relapses, and can even receive more than one transplant. The expected number of times a subject visits a certain state (in this example, the expected number of remissions or expected number of relapses etc.) can be estimated.

If a state can only be visited once, such as dementia, cardiovascular disease, and arthritis, then this quantity is also referred to as the remaining lifetime risk of the condition which can be interpreted as the probability of acquiring the condition in the future for those currently condition-free. Jack et al. investigated the association between amyloid level, sex, APOE genotype, and incident dementia and mortality among individuals without dementia [15]. They found that the remaining lifetime risk of dementia, i.e., the risk of dementia given that the individual is not demented at a certain age, varied considerably across groups of subjects defined by APOE genotype, sex, and amyloid level. Among patients with characteristics most likely to develop Alzheimer’s disease, women have a higher remaining lifetime risk than men. Specifically, remaining lifetime risk of dementia at age 65 for females with APOE ε4 and moderate amyloid levels was 58% (95% confidence interval [CI] 52–65%) compared to males with the same APOE and amyloid status where their lifetime risk is 44% (95% CI 35–53%). Among those with APOE ε4 and high levels of amyloid, the remaining lifetime risk at age 65, increases to 74% (95% CI 65–84%) for women compared to 62% (95% CI 52–73%) for men. The remaining lifetime risks at 65 reflect how likely individuals will experience dementia in the remainder of their lifetime given that they are dementia-free at age 65. In this example, although the rates of dementia were not necessarily higher in women compared to men, women have a higher remaining lifetime risk because women live longer than men, on average. The remaining lifetime risks provide a complementary perspective to the hazard ratios which represent the ratio of the rates of dementia in individuals with an exposure relative to a reference group. Both measures are meaningful and are complimentary to each other. Using both provide a fuller picture of the disease process.

#### Cox-Type Regression for Multistate Models

The risk measures obtained from the non-parametric Aalen-Johansen estimates as discussed above are unadjusted for other covariates. In settings where adjustment for potential confounders are needed, a Cox-type regression analysis for multistate data can be conducted to simultaneously model the association between the treatment or exposures and all transitions of interest. Please note that, traditional Cox proportional hazards model, ignoring the intermediate events of a multistate model, produces a single “average” relative risk of the terminal event. The work by Allen et al. in nonalcoholic fatty liver disease incidence and its impact on metabolic comorbidity burden and death show an example of the insights that can be obtained by fitting a Cox regression for a multistate model [9]. Because NAFLD and metabolic comorbidities (i.e., diabetes mellitus, hypertension, and hyperlipidemia) have intertwined pathophysiology, the independent impact of NAFLD on death may vary with the number of metabolic comorbidities a subject developed through the course of the disease. Allen et al. identified the association of NAFLD and an increased risk of developing metabolic comorbidities after adjusting for age and sex; and that the independent association of NAFLD and mortality decreases as the number of metabolic conditions increases [9]. Specifically, for subjects with no metabolic comorbidities, NAFLD was associated with a twofold increase in mortality risk (Relative Risk: 2.16, 95% CI: 1.41–3.31); whereas for subjects with three metabolic comorbidities, the association between NAFLD and mortality reduced to a relative risk 1.08 (95% CI: 0.89–1.30). These insights would not be easily untangled using traditional survival analysis approaches.

It is worth noting that, when applying multistate model to competing risks data, the transition intensity (i.e., the hazard of transition from a state to another) is indeed the cause-specific hazard. The sub-distribution hazard, developed by Fine and Gray, does not have a simple direct relationship with the measures described above for multistate model [16].

## Conclusions

Multistate models provide a flexible framework for analysis of time-to-event data. Multistate models encompass a wide range of models from the simplest 2-state mortality model to more complex models that include multiple states with repeated visits. However, multistate models are less known than other survival analysis methods including the Kaplan–Meier estimator and the Cox proportional hazards models. In this manuscript, we introduced common terminologies and quantities that can be estimated using multistate models. Examples were included to illustrate and highlight the utility of multistate model.

In any study with time-to-event data, a sketch of a state space including the events and the transitions that gave rise to the data is a useful starting point. The state space can then be refined to represent a multistate model that appropriately address the scientific question of interest. When consider a multistate model, it is important to balance between the model’s complexity and its’ interpretation. A multistate model should only include the states and the transitions necessary to answer the scientific questions of interest; additionally, each transition needs to include a sufficient number of patients to allow reliable estimation of the model.

Readers may have noticed that different time scales were used in our examples. Le-Rademacher et al. used the time “since randomization” while Jack et al. and Allen et al. both used “age” as the time scale. The time scale to use depends on the research questions and the intended interpretation. Since data in Le-Rademacher et al. is from a clinical trial where patients started treatment soon after randomization and the question of interest for the trial was the effect of trial treatments on clinical outcomes, randomization represents a natural and appropriate starting time for this study. On the other hand, both Jack et al. and Allen et al. evaluated the natural history and progression of age-related conditions, age serves as an appropriate time scale which provides a natural interpretation of the analysis results, i.e., the probability of being in a certain state at a certain age, how long the participants will stay in the state given other covariates, and remaining lifetime risk of a condition, such as dementia. Choosing the appropriate time scale is an important consideration for multistate models.

Using counting process, multistate models can easily accommodate data with left truncation [4•]. Both Jack et al. and Allen et al. used “age” as the time scale which are examples of multistate models with left truncated data. Additionally, since multistate analytical methods are extensions of classical survival analysis methods based on counting process, statistical measures from multistate models have similar interpretations and limitations as their counterparts in classical survival analysis [17•, 18•, 19•]. Specifically, the challenges of using the hazard ratio from traditional Cox model in causal inference apply to the hazard ratio derived from Cox-type regression model from multistate model. Quantities of absolute risks such as time spent in a state and probability of being in a state may be more appropriate for causal inference [20, 21].

Multistate models is an analytical approach that may shed light on complex disease process. However, they are still susceptible to potential collider stratification bias and confounding effect as other analytical approaches. Care must be taken to minimize these potential issues, especially for observational studies.

## Supplementary Information

Below is the link to the electronic supplementary material.

Supplementary file1 (DOCX 130 KB)

Supplementary file2 (DOCX 68 KB)
