# Supplementary material for: The Utility of Multistate Models: A Flexible Framework for Time-to-Event Data
Source: Curr Epidemiol Rep. 2022 Jun 29;9(3):183–9. doi: 10.1007/s40471-022-00291-y (PMC9392702; doi:10.1007/s40471-022-00291-y)
Supplement: Supplementary file 1 — Supplementary file1 (DOCX 130 KB) [file 40471_2022_291_MOESM1_ESM.docx]

Appendix: Supplemental examples

Terry Therneau, Fang-Shu Ou, Jennifer Le-Rademacher

# Introduction

This supplemental material contains R code which can be used to create results in the manuscript. An overall message of the manuscript is that multistate models are easily accessible using modern software. These examples all use the the survival library within R (version 3.1 or higher). No claim is made that R is the best or only software for this, or even that within R the survival library is best or easiest. The point is to show a reasonably simple example that works. One small advantage of the survival library is that it is part of the recommended set for R, so it will always be available.

# State space figures

This reproduces Figure 1 of the manuscript, with 4 state space figures. With the proliferation of interest in directed acyclic graphs (DAG) there are many routines in R to draw box and arrow figures. The *statefig* routine from the survival package was designed to produce “good enough” figures while being very easy to use.

If there are k states, create a k by k matrix of 0/1 values, where the i,j element is 1 if there is an arrow from state i to state j. The row/column labels of the matrix are used for the state labels. If the first argument of *statefig* is a matrix, then the states are laid out one row at a time. If the first argument of *statefig* is a vector (a, b, c), this tells the routine to put the first ‘a’ states in column 1 of the figure, then next ‘b’ in column 2, etc.

oldpar <- par(mar=c(.1, .1, .1, .1), mfrow=c(2,2))
# first figure (simple mortality)
states <- c("Alive","Dead")
cmat1 <- matrix(0,nrow=2,ncol=2, dimnames=list(states,states))
cmat1[1,2] <- 1
statefig(matrix(2,1,1), cmat1)

# second figure (competing risk)
states <- c("Alive","Death cause 1","Death cause 2","Death cause 3")
cmat2 <- matrix(0,nrow=4,ncol=4, dimnames=list(states,states))
cmat2[1,2:4] <- 1
statefig(c(1,3), cmat2)

# third figure (illness-death)
states <- c("Health","Illness","Death")
cmat3 <- matrix(0,nrow=3,ncol=3, dimnames=list(states,states))
cmat3[1,2] <- 1
#cmat3[2,1] <- 1 #leave this one off
cmat3[,3] <- 1 # all cmat3 to death
statefig(c(1,2), cmat3, offset=.02)

# fourth figure (NAFLD)
states <- c("0 MC", "1 MC", "2 MC", "3 MC", "Death")
cmat4 <- matrix(0, 5,5, dimnames= list(states, states))
cmat4[1,2] <- cmat4[2,3] <- cmat4[3,4] <- 1
cmat4[-5,5] <- 1
statefig(c(4,1), cmat4)


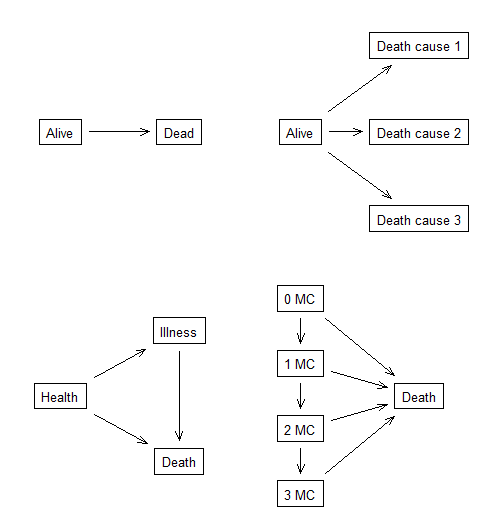


A collection of state space

par(oldpar)

Because the state space is central to these models, our suggestion is to start any analysis by printing out a copy to display prominently. Note that there are further options for colors and line types, though they are rarely needed for this purpose. One can use values less than or greater than 1 in the matrix to produced curved arrows.

# Creating the data

As anyone who has done analysis knows, creation of the proper data set is often 2/3 or more of the actual work in producing an analysis. For multistate models the survival library expects to have a counting process data set of the following form:

- Each subject occupies one or more rows, along with an identifier variable which identifies which rows belong to a given subject.
- Each row has a trio of variables (time1, time2, state), and contains subject information over the time interval (time1, time2], along with the result of a transition (state), if any, at time2. Covariate values in that row are the values which apply to the subject over that time interval. The state variable is a factor with levels for each state, along with one for “no transition occurred at this time”. The “no transition” outcome must be the first level of the factor.
- Each subject must describe an actual path through time: no overlapping intervals (one patient cannot be at two places/states at once), and no gaps (one patient must be somewhere, i.e. at a state). There are rare exceptions to the second rule which we will not pursue.
- Often there will be a “current state” variable; it is necessary, for instance, if everyone does not start in the same state.

Counting process data have become quite a common way to deal with time dependent covariates in the ordinary Cox model, so data sets like this should be familiar. The major change is the use of a state variable rather than a simple 0/1 status. The (time1, time2) form has been available for many years, so of course there are several ways to build such a data set. Below we create the data set for the myeloid analysis. The base data in this case has one record per subject with the baseline covariates (treatment and sex) along with the times to complete response (CR), stem cell transplat (SCT), relapse, and death. The code below demonstrates how the data is created and, most importantly, a call to the *survcheck* routine. The *survcheck* routine gives useful information on the data set, but more importantly, it checks for consistency with the rules mentioned above.

# details and explanations for all the steps below can be found in the
# vignettes for the survival package
tied <- with(myeloid, (!is.na(crtime) & !is.na(txtime) & crtime==txtime))
# one subject has CR on the same day as transplant.
# Move the CR one day earlier and use a temporary data set
tdata <- myeloid
tdata$crtime[tied] <- tdata$crtime[tied] -1
# tmerge does most of the work
mdata <- tmerge(tdata[,1:3], tdata, id=id, death= event(futime, death),
 sct = event(txtime), cr = event(crtime),
 relapse = event(rltime),
 priorcr = tdc(crtime), priortx = tdc(txtime),
 priorrl= tdc(rltime))

# create the overall state variable (called event)
temp <- with(mdata, cr + 2*sct + 4*relapse + 8*death)
mdata$event <- factor(temp, c(0,1,2,4,8),
 c("none", "CR", "SCT", "relapse", "death"))

# create a competing risk endpoint, first of CR or death without CR
# Since in this case each subject has only one row, we can just add new
# variables (ctime, cstat) to the myleloid data, no need for (time1, time2)
myeloid$ctime <- with(myeloid, ifelse(is.na(crtime), futime, crtime))
temp <- with(myeloid, ifelse(is.na(crtime), 2*death, 1))
myeloid$cstat <- factor(temp, 0:2, c("censor","CR", "death"))

# and an endpoint that ignores SCT
temp1 <- mdata$event
temp1[temp1=="SCT"] <- "none"
mdata$event2 <- temp1

# Data check, and count up the observed transitions
survcheck(Surv(tstart, tstop, event) ~ 1, data = mdata, id = id)

Call:
survcheck(formula = Surv(tstart, tstop, event) ~ 1, data = mdata,
 id = id)

Unique identifiers Observations Transitions
 646 1689 1364

Transitions table:
 to
from CR SCT relapse death (censored)
 (s0) 443 106 13 55 29
 CR 0 159 168 17 110
 SCT 11 0 45 149 158
 relapse 0 99 0 99 28
 death 0 0 0 0 0

Number of subjects with 0, 1, ... transitions to each state:
 count
state 0 1 2 3 4
 CR 192 454 0 0 0
 SCT 282 364 0 0 0
 relapse 420 226 0 0 0
 death 326 320 0 0 0
 (any) 29 201 174 153 89

There are 1,364 transitions among 646 subjects. Everyone starts in the same state, so it was not necessary to create a “current state” variable. Lacking it, the routine uses (s0) as the label for the common starting state. The conceptual model for this study was initial therapy, CR, SCT, relapse, death; the initial therapy leads to complete response (CR), which in turn sets the stage for stem cell transplant (SCT). Nevertheless, we see that 106 subjects went directly to SCT, without CR; a few even saw their first CR after SCT. Real data is always more complex than planned.

The second table shows how many subjects has 0, 1, etc of each event type. By design, the study only collected the first CR for any subject, the first SCT, etc.; in this aspect the study data is as planned. There were 29 subjects who went from (s0) directly to censoring, i.e., they had no events, and another 89 who visited all five states.

We can also check out the alternate endpoint. There are 0 SCT using the crstat variable, and the state does not appear in the *survcheck* tables.

table(mdata$event2)

none CR SCT relapse death
 689 454 0 226 320

survcheck(Surv(tstart, tstop, event2) ~1, data = mdata, id = id)

Call:
survcheck(formula = Surv(tstart, tstop, event2) ~ 1, data = mdata,
 id = id)

Unique identifiers Observations Transitions
 646 1689 1000

Transitions table:
 to
from CR relapse death (censored)
 (s0) 454 20 102 70
 CR 0 206 50 198
 relapse 0 0 168 58
 death 0 0 0 0

Number of subjects with 0, 1, ... transitions to each state:
 count
state 0 1 2 3
 CR 192 454 0 0
 relapse 420 226 0 0
 death 326 320 0 0
 (any) 70 303 122 151

# Aalen-Johansen curves

Now draw four different curves. The *survfit* function computes Aalen-Johansen curves (of which the Kaplan-Meier is a special case). As is often the case, the annotations to make the curve “pretty” for the manuscript take up most of the room: xmax, xscale, xlab, ylab, ylim, lty, legend, and added text.

# Simple survival.
msurv1 <- survfit(Surv(tstart, tstop, event=="death") ~ trt, mdata)
# plot(msurv1) # simple plot
# Use the fun argument to plot deaths instead of survival
plot(msurv1, fun="event", xscale= 30.5, xmax=732, lty=1:2,
 xlab="Months Since Randomization", ylab="Probability of Death",
 ylim=c(0, 0.8))
text(c(370, 520), c(.42, .24), c("A", "B"))


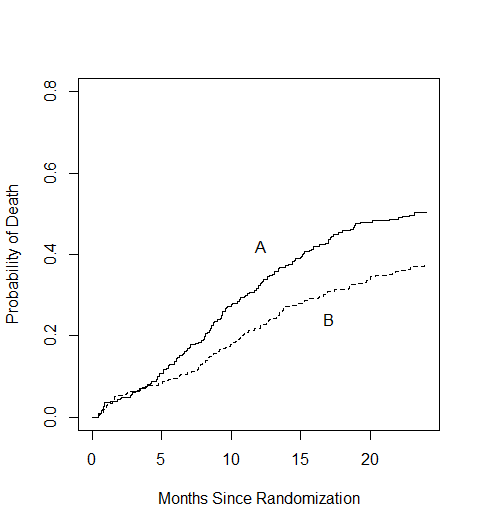


The competing risks plot, which uses the simple data set.

# Competing risk of CR and death without CR.
msurv2 <- survfit(Surv(ctime, cstat) ~ trt, myeloid, id=id)
dim(msurv2) # a matrix of curves

strata states
 2 3

# rows=treatment, cols= entry (randomization), CR, death

# don't plot the entry curve, as it is redundant
plot(msurv2[,2:3], xscale= 30.5, xmax=732, lty=1:2,
 xlab="Months Since Randomization", ylab="Probability in State",
 ylim=c(0,0.8))
text(c(450, 450), c(.64, .03), c("CR", "Death without CR"))
legend(400,.5, c("A", "B"), lty=1:2, bty='n')


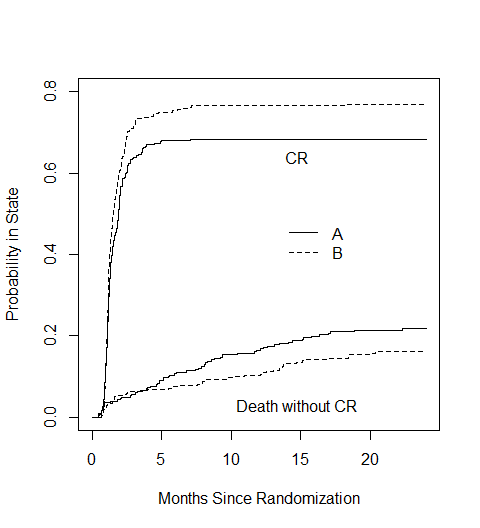


The plot to understand duration of CR. Subjects can transition out of CR to relapse or death, but SCT is ignored.

msurv3 <- survfit(Surv(tstart, tstop, event2) ~ trt, data= mdata, id=id)
dim(msurv3) # all 5 states appear in the result, but SCT curve is 0

strata states
 2 5

# The plot in the manuscript. Overlay the CR curves from msurv2 (competing risk), and the "still in CR" curves from msurv3.
plot(msurv2[,2], xscale= 30.5, xmax=732, lty=1:2,
 xlab="Months Since Randomization", ylab="Probability of CR", ylim=c(0, 0.8))
lines(msurv3[,2], xscale= 30.5, xmax=732, lty=1:2)
text(c(400, 500), c(.73, .1), c("Ever CR", "Sustained CR"))
legend(400,.65, c("A", "B"), lty=1:2, bty='n')


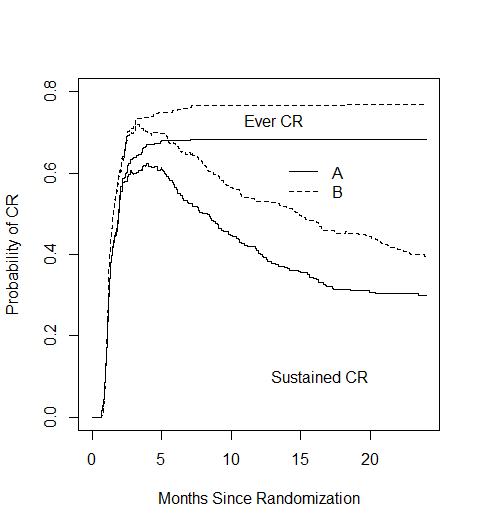


And finally, the figure with all the states, extended out to 48 months. The manuscript did not use color, but it helps on a screen. The only curve that always rises is death, as expected.

msurv4 <- survfit(Surv(tstart, tstop, event) ~ trt, mdata, id=id)
plot(msurv4, xscale= 30.5, xmax=1460, lty=1:2, col=c(1,1,2,2,3,3,4,4),
 xaxt= 'n', xlab="Months Since Randomization",
 ylab="Probability in State", ylim=c(0, 0.8))
mm <- c(0, 6, 12, 24, 36, 48)
axis(1, mm*30.5, mm)
text(c(1277, 1320, 1360, 1215), c(.50, .35, .12, .005), col=c(4,2,1,3),
 c("Death", "SCT", "CR", "Relapse"))


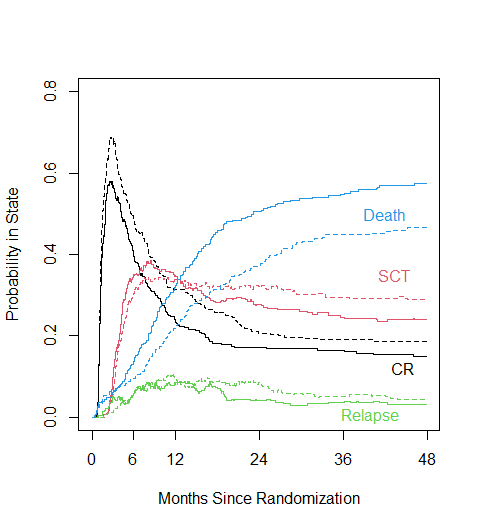


# Multistate (proportional) hazards models

## Myeloid

When the data is correctly set up (passes *survcheck*) then the *coxph* routine can be used to easily fit either an ordinary proportional hazards (Cox) model or a multistate hazards model. Below this is done for the three curves investigated above.

mfit1 <- coxph(Surv(tstart, tstop, event=="death") ~ trt + sex, mdata)
mfit1

Call:
coxph(formula = Surv(tstart, tstop, event == "death") ~ trt +
 sex, data = mdata)

 coef exp(coef) se(coef) z p
trtB -0.3582 0.6989 0.1129 -3.174 0.00151
sexm 0.1150 1.1219 0.1128 1.020 0.30782

Likelihood ratio test=10.56 on 2 df, p=0.005093
n= 1689, number of events= 320

mfit2 <- coxph(Surv(ctime, cstat) ~ trt + sex, id=id, myeloid)
mfit2

Call:
coxph(formula = Surv(ctime, cstat) ~ trt + sex, data = myeloid,
 id = id)


1:2 coef exp(coef) se(coef) robust se z p
 trtB 0.22797 1.25604 0.09457 0.09476 2.406 0.0161
 sexm 0.07436 1.07720 0.09465 0.09512 0.782 0.4344


1:3 coef exp(coef) se(coef) robust se z p
 trtB -0.1246 0.8829 0.1851 0.1809 -0.689 0.4910
 sexm 0.4594 1.5831 0.1845 0.1815 2.531 0.0114

 States: 1= (s0), 2= CR, 3= death

Likelihood ratio test=13.18 on 4 df, p=0.01041
n= 646, number of events= 573

The fit *mfit1* is an ordinary Cox model and shows the effect of treatment on overall death rate. The second, multistate model fits separate hazard models for the two transitions of randomization:CR and randomization:death (competing risk). It shows that treatment B has a 1.26 fold higher CR rate, while males have a 1.58 fold higher rate of death before CR. Male sex has little effect on the CR rate and treatment has little effect on the rate of death without CR. This is, again, evidence that CR is an important component of the overall superiority of treatment B.

The predicted competing risk curves from the fit are fairly simple. As with any proportional hazards model, one must specify covariate values for each desired curve.

# four combinations
dummy <- data.frame(trt=c("A", "A", "B", "B"), sex= c("f","m", "f", "m"))
mfit2.surv <- survfit(mfit2, newdata=dummy)
plot(mfit2.surv, col=1:2, lty= c(1,1,2,2), xscale= 30.5, xmax=720,
 xlab="Months Since Randomization", ylab= "Probability in State",
 ylim=c(0, 0.8))
text(c(615, 615), c(.72, .08), c("CR", "Death without CR"))
legend(350, .55, c("Female, A", "Female B", "Male, A", "Male B"),
 col=c(1,1,2,2), lty=c(1,2,1,2), bty='n')


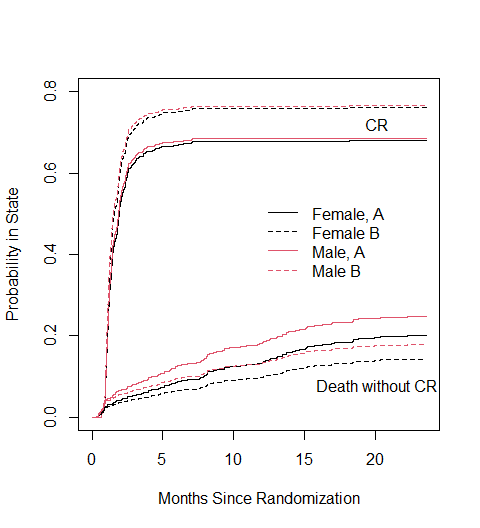


When there are multiple transitions, the effects of each hazard on the outcome are intertwined, and it can be difficult to visualize the final impacts. In the above, the estimated hazard rate for death without CR differs by much more between males and females (Hazard ratio [HR]=1.58) than between treatment A and B (HR=1.07). Yet the differences between *realized* non-CR death at 24 months for males vs females and A vs B are nearly the same, due to the higher CR rate for B, which decreases the population of B subjects at risk. With multistate models, plots of the absolute risk are a necessary companion to the estimates of relative risk.

## Fatty liver disease (NAFLD)

The NAFLD data is represented as 3 data sets, nafld1 has one observation per subject containing basline information (age, sex, etc.), nafld2 has information on repeated laboratory tests, e.g. blood pressure, and nafld3 has information on yes/no endpoints. The second and third data sets are in long form, i.e. each row has the subject id, date, type of result, and result. Start by building an analysis data set using nafld1 and nafld3 Much more detail about this process is in the main vignette of the survival package.

ndata <- tmerge(nafld1[,1:8], nafld1, id=id, death= event(futime, status))
ndata <- tmerge(ndata, subset(nafld3, event=="nafld"), id,
 nafld= tdc(days))
ndata <- tmerge(ndata, subset(nafld3, event=="diabetes"), id = id,
 diabetes = tdc(days), e1= cumevent(days))
ndata <- tmerge(ndata, subset(nafld3, event=="htn"), id = id,
 htn = tdc(days), e2 = cumevent(days))
ndata <- tmerge(ndata, subset(nafld3, event=="dyslipidemia"), id=id,
 lipid = tdc(days), e3= cumevent(days))
ndata <- tmerge(ndata, subset(nafld3, event %in% c("diabetes", "htn",
 "dyslipidemia")),
 id=id, comorbid= cumevent(days))

# The current state for each subject, at the start of an interval
ndata$cstate <- with(ndata, factor(diabetes + htn + lipid, 0:3,
 c("0mc", "1mc", "2mc", "3mc")))
temp <- with(ndata, ifelse(death, 4, comorbid))
#the final transition, if any, at the end of each interval
ndata$event <- factor(temp, 0:4,
 c("censored", "1mc", "2mc", "3mc", "death"))
ndata$age1 <- ndata$age + ndata$tstart/365.25 # analysis on age scale
ndata$age2 <- ndata$age + ndata$tstop/365.25

# Check the data
check1 <- survcheck(Surv(age1, age2, event) ~ nafld + male, data=ndata,
 id=id, istate=cstate)
check1

Call:
survcheck(formula = Surv(age1, age2, event) ~ nafld + male, data = ndata,
 id = id, istate = cstate)

Unique identifiers Observations Transitions
 17549 22683 6186

Transitions table:
 to
from 1mc 2mc 3mc death (censored)
 0mc 1829 70 4 263 5705
 1mc 0 1843 28 243 4567
 2mc 0 0 1048 417 3687
 3mc 0 0 0 441 2220
 death 0 0 0 0 0

Number of subjects with 0, 1, ... transitions to each state:
 count
state 0 1 2 3 4
 1mc 15720 1829 0 0 0
 2mc 15636 1913 0 0 0
 3mc 16469 1080 0 0 0
 death 16185 1364 0 0 0
 (any) 12733 3673 938 183 22

The transitions table tells us that the transition matrix is not quite what is shown in Figure 1d of the manuscript. It actually looks like the figure below:

states <- c("No comorbidity", "1 comorbidity", "2 comorbidities",
 "3 comorbidities", "Death")
cmat <- matrix(0, 5,5)
cmat[,5] <- 1
cmat[1,2] <- cmat[2,3] <- cmat[3,4] <- 1
cmat[1,3] <- cmat[2,4] <- 1.6
cmat[1,4] <- 1.6
dimnames(cmat) <- list(states, states)
statefig(cbind(4,1), cmat, alty=c(1,2,1,2,2,1,1,1,1,1,1))


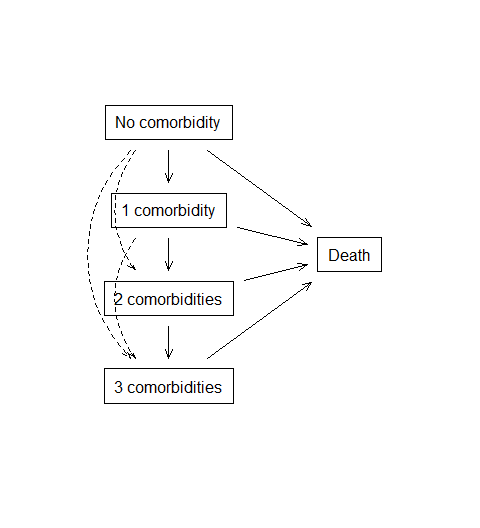


There are 70 subjects for instance who skip directly from 0 to 2 comorbidities; a reflection of the fact that some subject are evaluated infrequently; there are 4 who appear to go directly from 0 to 3. The number of subjects who traverse each of the dotted arrows is small. The authors decided to force the coefficients for those transitions to be identical to more prevalent ones, as a way to stabilize the model fit. This requires a more complicated *coxph* call that adds the constraints.

nfit1 <- coxph(list(Surv(age1, age2, event) ~ nafld + male,
 "0mc":c("1mc", "2mc", "3mc") ~ nafld+ male / common,
 "1mc":c("2mc", "3mc") ~ nafld + male / common),
 data=ndata, id=id, istate=cstate)
round(coef(nfit1, matrix= TRUE), 3)

1:2 1:3 2:3 1:4 2:4 3:4 1:5 2:5 3:5 4:5
nafld 0.915 0.915 0.521 0.915 0.521 0.485 0.634 0.529 0.552 0.063
male 0.179 0.179 0.246 0.179 0.246 0.149 0.430 0.472 0.361 0.161
attr(,"states")
[1] "0mc" "1mc" "2mc" "3mc" "death"

In the above fit there is a common coefficient for all 3 transitions from 0 MC to a 1-3 comorbidities, as reflected in the coefficients table, and likewise for a transition from 1 MC to either 2 or 3 MC. The presence of NAFLD has a major influence (HR=2.5) on leaving the 0 MC state, slightly less on leaving 1 MC (HR=1.7), and somewhat less on 2MC:3MC (HR=1.6). Males gain comorbidities faster than females and have higher death rates, neither of which is a surprise. The fit was done using age as the underlying time scale, so all of the hazard ratios are in comparison to subjects who were the same age. The probability in state curves can be obtained using the *survfit* function, as before. We create conditional curves, for hypothetical subjects who are alive at age 60, and currently with 0 comorbidities. (In clinical practice, one would want to generate the appropriate curve, in real time, for an actual patient using their current age and state).

dummy <- data.frame(nafld= c(0,1, 0, 1), male= c(0, 0, 1, 1)) # four subjects
nsurv0 <- survfit(nfit1, newdata=dummy, start.time= 60, p0 =c(1,0,0,0,0))
dim(nsurv0)

data states
 4 5

plot(nsurv0[1:2,], lty=1:2, lwd=2, col=rep(1:5, each=2),
 xlab="Age", ylab="P(state), females")
legend(75, 1, c("0 MC", "1 MC", "2 MC", "3 MC", "death"), lty=1, col=1:5,
 lwd=2, bty='n')


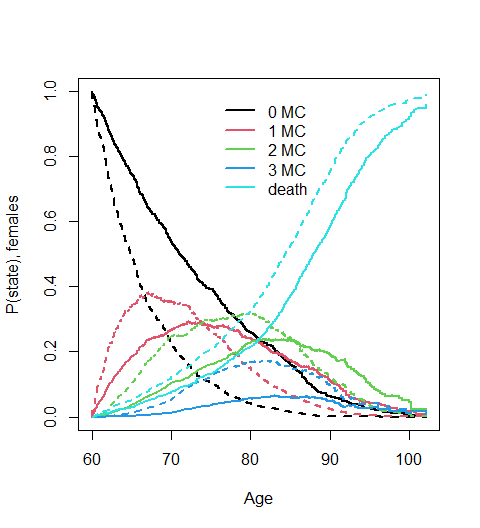


The plot is very busy, containing 10 predicted curves from the fitted model; dashed lines for a 60 year old with NAFLD and solid for someone without. The curves for the 0 MC and death states are easy to pick out, since the first is monotone down and second monotone up. As a simpler summary, Allen focused on the restricted mean time in state (RMST or sojourn time), up to age 100, which yields a single number for each scenario. The resulting values can be interpreted as the number of years, out of the next 40, that a subject is expected to be in each of the states.

# print(nsurv0, rmean = 100) # simple printout
temp <- summary(nsurv0, rmean=100)
rmean <- matrix(temp$table[,"rmean"], ncol=5,
 dimnames= list(c("Female, control", "Female NAFLD",
 "Male control", "Male NAFLD"), nsurv0$states))

round(rmean, 1)

0mc 1mc 2mc 3mc death
Female, control 13.3 6.7 5.4 1.3 13.4
Female NAFLD 6.6 6.3 6.7 3.3 17.0
Male control 11.2 5.7 5.2 1.5 16.4
Male NAFLD 5.4 5.1 6.0 3.6 19.8

par(mar= c(5,7,1,1)) # more space in left margin
barplot(t(rmean[c(2,1,3,4),]), col=c(1,2,3,4,0), horiz=TRUE, las=1,
 border=NA, xlab="Years", xlim=c(0,30))


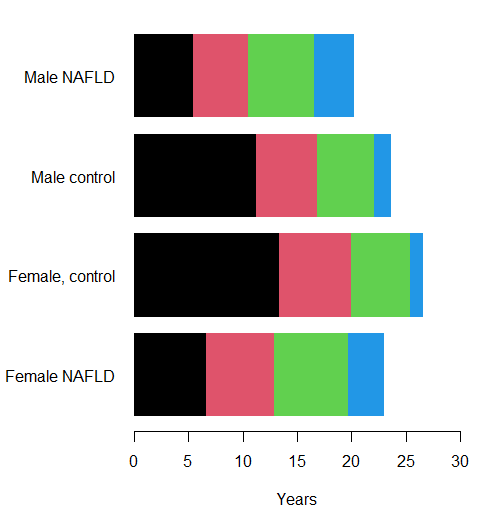


The center two bars are male and female controls. We see that females live longer and spent more time in the 0 MC state than males. Comparing males with and without NAFLD, males with NAFLD have a shorter life and more total years of that remaining life will be spent in the 2 and 3 comorbidity states. Same pattern is observed comparing females with and without NAFLD.

## Age and dementia

The data set for the Mayo Clinic Study of Aging is not publicly available.
